# Duration of exposure to multiple antibiotics is associated with increased risk of VRE bacteraemia: a nested case-control study

**DOI:** 10.1093/jac/dky075

**Published:** 2018-03-13

**Authors:** Theodore Gouliouris, Ben Warne, Edward J P Cartwright, Luke Bedford, Chathika K Weerasuriya, Kathy E Raven, Nick M Brown, M Estée Török, Direk Limmathurotsakul, Sharon J Peacock

**Affiliations:** 1 Department of Medicine, University of Cambridge, Cambridge, UK; 2 Public Health England, Clinical Microbiology and Public Health Laboratory, Cambridge, UK; 3 Cambridge University Hospitals NHS Foundation Trust, Cambridge, UK; 4 Mahidol-Oxford Tropical Medicine Research Unit, Mahidol University, Bangkok, Thailand; 5 London School of Hygiene & Tropical Medicine, Keppel Street, London, UK

## Abstract

**Background:**

VRE bacteraemia has a high mortality and continues to defy control. Antibiotic risk factors for VRE bacteraemia have not been adequately defined. We aimed to determine the risk factors for VRE bacteraemia focusing on duration of antibiotic exposure.

**Methods:**

A retrospective matched nested case-control study was conducted amongst hospitalized patients at Cambridge University Hospitals NHS Foundation Trust (CUH) from 1 January 2006 to 31 December 2012. Cases who developed a first episode of VRE bacteraemia were matched 1:1 to controls by length of stay, year, specialty and ward type. Independent risk factors for VRE bacteraemia were evaluated using conditional logistic regression.

**Results:**

Two hundred and thirty-five cases were compared with 220 controls. Duration of exposure to parenteral vancomycin, fluoroquinolones and meropenem was independently associated with VRE bacteraemia. Compared with patients with no exposure to vancomycin, those who received courses of 1–3 days, 4–7 days or >7 days had a stepwise increase in risk of VRE bacteraemia [conditional OR (cOR) 1.2 (95% CI 0.4–3.8), 3.8 (95% CI 1.2–11.7) and 6.6 (95% CI 1.9–22.8), respectively]. Other risk factors were: presence of a central venous catheter (CVC) [cOR 8.7 (95% CI 2.6–29.5)]; neutropenia [cOR 15.5 (95% CI 4.2–57.0)]; hypoalbuminaemia [cOR 8.5 (95% CI 2.4–29.5)]; malignancy [cOR 4.4 (95% CI 1.6–12.0)]; gastrointestinal disease [cOR 12.4 (95% CI 4.2–36.8)]; and hepatobiliary disease [cOR 7.9 (95% CI 2.1–29.9)].

**Conclusions:**

Longer exposure to vancomycin, fluoroquinolones or meropenem was associated with VRE bacteraemia. Antimicrobial stewardship interventions targeting high-risk antibiotics are required to complement infection control procedures against VRE bacteraemia.

## Introduction

Over the last 20 years, VRE have emerged as a major cause of healthcare-associated bacteraemia, disproportionally affecting immunocompromised and critically ill patients.^1^*Enterococcus faecium* has become responsible for most VRE infections following the global dissemination of a hospital-adapted lineage.^2^ VRE bacteraemias are associated with increased costs of care, length of stay and mortality compared with bactaraemias caused by vancomycin-susceptible enterococci (VSE).^3^^,^^4^ In contrast to other healthcare-associated infections, rates of VRE bacteraemia have failed to decline in response to a host of generic infection control interventions in different healthcare settings^5–7^ and are even increasing in some countries.^8^ Consequently, the identification of modifiable risk factors for VRE bacteraemia remains a priority.

Gut carriage of VRE is a major risk factor for VRE bacteraemia. Bloodstream infection may be preceded by high levels of VRE carriage in the gut.^9^ In recipients of allogeneic stem cell transplants, this is observed in conjunction with loss of microbiota diversity (particularly anaerobes), a state termed enterococcal dominance.^10^ Exposure to a range of antibiotics increases susceptibility to VRE intestinal colonization and progression to high-level carriage and bacteraemia, although the effect of individual antibiotics varies at each step of this sequence of events.^11^ The rate of progression from carriage to invasive infection is also affected by the comorbidities of the patient population.^12^ Length of stay, adherence to infection control procedures and proximity to VRE-colonized patients or a contaminated environment are additional modifiable factors that affect the risk of VRE colonization.^13^

A number of studies have identified risk factors for VRE bacteraemia, including haematological malignancy, renal insufficiency, acute severity of illness, immunosuppression/neutropenia, gastrointestinal disease or procedures and modifiable factors such as antibiotic exposure.^14–24^ Vancomycin is the antibiotic most commonly implicated, but not all studies agree on its role. Lastly, few studies have quantified the effect of cumulative exposure to individual antibiotics.^15^^,^^22^^,^^23^

The aim of this study was to identify modifiable risk factors for VRE bacteraemia, in particular antibiotic exposure, using a nested case-control study design in a centre with high rates of VRE endemicity.

## Methods

### Study setting, design and participants

A retrospective matched nested case-control study was conducted amongst hospitalized patients at Cambridge University Hospitals NHS Foundation Trust (CUH) in the UK from 1 January 2006 to 31 December 2012. This tertiary referral teaching hospital has 1170 beds, 340 000 occupied bed-days per year and a range of specialties including hepatology and hepatobiliary surgery, solid organ transplantation (kidney, liver, pancreas and small bowel/multivisceral), adult haematopoietic stem cell transplantation, paediatric haemato-oncology, and general and neurocritical ICUs. CUH has reported the highest number of VRE bacteraemias in England in the national mandatory surveillance scheme from 2003 to 2012 (426/6246 or 7% of national total out of 161 hospital Trusts). An active antimicrobial stewardship programme was in place throughout the duration of the study, including prescribing guidelines and regular antimicrobial rounds. Infection control practices targeting VRE did not change during the study period; however, a line-care bundle was implemented during 2006 and a deep-clean programme in 2007. Vancomycin and teicoplanin susceptibility was determined by disc diffusion using BSAC breakpoints (http://www.bsac.org.uk/wp-content/uploads/2012/02/BSAC-Susceptibility-testing-version-143.pdf). Cases and controls were identified using the diagnostic laboratory information system and the hospital electronic database, respectively. Cases were consecutive inpatients with their first episode of VRE bacteraemia during the study period. Patients with presumed contaminated blood cultures (single positive sets not necessitating the use of targeted antibiotic therapy for symptom and bacteraemia resolution at the clinicians’ discretion) were excluded (Table S1, available as Supplementary data at *JAC* Online). Controls were matched to cases in a 1:1 ratio for the following: (i) duration of stay (matched to cases based on time from admission to day that positive blood culture was taken); (ii) year of admission; (iii) specialty; and (iv) ward type defined as general adult, adult ICU or paediatric ward. Specialty and ward type were treated as time-varying variables and matched at the day of the bacteraemia. Matching for year of admission was chosen to minimize potential confounding that may arise owing to changes in antimicrobial prescribing or infection control practices during the study period. Matching for specialty and ward type was used to account for underlying comorbidities that predispose to VRE infections and for changes in local unit VRE prevalence. Cases could serve as controls before becoming a case and controls could serve as controls more than once.^25^

### Covariates

Demographic, epidemiological and clinical information were selected for inclusion based on a literature review of risk factors for VRE bacteraemia and extracted from paper and electronic patient records. These included duration of hospital stay and prior ICU stay at CUH up to the point of matching, in-patient transfer from another hospital at the start of the current admission and cumulative length of stay in all wards and high-risk wards at CUH over the year prior to current admission. High-risk wards were those associated in the literature with increased risk of VRE colonization and invasive disease (adult and paediatric haemato-oncology, solid organ transplant, nephrology, hepatology and ICU).^26^ Mortality at 30 days was determined from hospital records or from an online national database (NHS Spine). Usage data for all antibacterial and antifungal agents (including treatment and prophylactic doses) were collected for 30 days prior to matching from paper local and referring hospital records and drug charts. Cut-offs for duration of antimicrobial exposure were chosen at 3 days and 7 days in line with current antimicrobial stewardship recommendations in which indication for continuing antibiotics should be reviewed at 48–72 h and prolonged courses beyond one week are discouraged in the absence of a clear indication.^27^

Clinical parameters and comorbidities recorded at the time of matching included use of gastric acid-suppressing medication; presence of a central venous catheter (CVC); neutropenia (neutrophil count <500 × 10^6^/L); immunosuppression (other than neutropenia); hypoalbuminaemia (albumin <30 g/dL); solid organ or haematological malignancy; solid organ or haematopoietic stem cell transplantation; liver cirrhosis; gastrointestinal disease; and hepatobiliary disease. VRE carriage was defined as growth of VRE from any clinical culture in the 12 months prior to matching. An additional list of candidate variables and definitions is available as Supplementary data.

### Statistical methods

Our primary analysis examined the association between exposure to antimicrobials and the subsequent development of VRE bacteraemia. We used univariable and multivariable logistic regression models conditioned on the matched variable to estimate conditional ORs (cORs) and 95% CIs for the association between independent factors and the development of VRE bacteraemia. Variables were evaluated in a multivariable model if differences between cases and controls on univariable analysis showed a *P* value <0.2. The final multivariable model was built using Hosmer and Lemeshow’s purposeful selection.^28^ Statistical analyses were performed using the Stata 12.1 software package (Stata Corp., USA).

### Ethics

The study was approved by the local Research Ethics Committee (reference 13/EE/0044) and by the CUH Research and Development Department (reference A092807) and the need for informed consent was waived.

## Results

**Figure 1.**
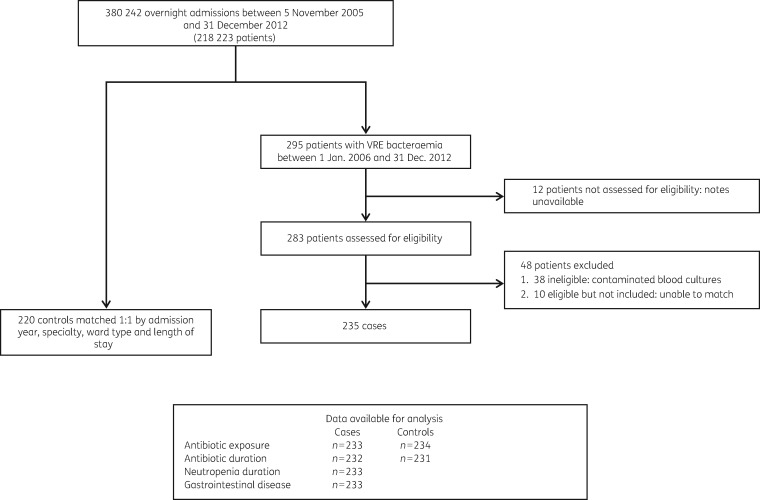
Selection of study population for nested case-control comparison of risk factors for VRE bacteraemia.

We identified 295 patients with 331 episodes of VRE bacteraemia from 1 January 2006 to 31 December 2012 (average annual incidence of 12.8/100 000 occupied bed-days) (Figure 1). These originated from a base of 218 223 patients that had 380 242 overnight admissions from 5 November 2005 to 31 December 2012. Twelve patients could not be assessed owing to missing paper records and 38 were excluded as their positive blood cultures were deemed contaminants by the treating doctors. Of the remaining 245 patients, 235 defined as cases were successfully matched to 220 controls. Eight cases also served as controls before becoming cases and seven controls were matched to cases more than once, resulting in 235 paired comparisons.

The demographic, clinical and microbiological characteristics of 235 cases and 220 controls are shown in Table 1. Comparison between the two groups confirmed effective matching for age, gender, speciality, ward type, year and length of stay. Thirty cases were younger than 16 years and, of the adult patients, 55 (27%) were located in an ICU at the onset of infection. VRE bacteraemia occurred in cases a median of 16 days following admission to CUH. *E. faecium* accounted for 91% and the VanA phenotype (resistance to both vancomycin and teicoplanin) for 87% of bacteraemias. The crude (all-cause) mortality at 30 days was higher in cases compared with controls (34% versus 13%). Only two deaths occurred in the paediatric population, both of which were cases.

**Table 1.** Demographic, clinical and microbiological characteristics of cases and controls

Characteristic	Cases: patients with VRE bacteraemia (*n *=* *235)	Controls: patients without VRE bacteraemia (*n *=* *220)	*P*
Age (years), median (IQR)	56.6 (39.0–66.7)	57.8 (42.2–69.5)	0.34
Male	145 (61.7)	127 (57.7)	0.47
Year of admission			
2005	2 (0.9)	2 (0.9)	NA
2006	34 (14.5)	33 (15.0)	
2007	42 (17.9)	39 (17.7)	
2008	30 (12.8)	29 (13.2)	
2009	39 (16.6)	33 (15.0)	
2010	31 (13.2)	29 (13.2)	
2011	25 (10.6)	25 (11.4)	
2012	32 (13.6)	30 (13.6)	
Ward at time of bacteraemia			
adult general	150 (63.8)	141 (64.1)	NA
adult ICU	55 (23.4)	53 (24.1)	
paediatric	30 (12.8)	26 (11.8)	
Time from admission to bacteraemia (cases) and matching (controls) (days), median (IQR)^a^	16 (9–31)	16 (8–31)	NA
Lead specialty type at time of matching^b^			
adult haematology	67 (28.5)	57 (25.9)	NA
adult oncology	4 (1.7)	4 (1.8)	
adult medicine	66 (28.1)	65 (29.6)	
adult solid organ transplant	31 (13.2)	31 (14.1)	
adult surgery	37 (15.7)	37 (16.8)	
paediatric haemato-oncology	30 (12.8)	26 (11.8)	
*Enterococcus faecalis*	17 (7.2)		
*E. faecium*	214 (91.1)		
Other enterococcal species^c^	4 (1.7)		
VanA	202/232 (87.1)		
Death within 30 days of matching	79 (33.6)	28 (12.7)	<0.001^d^

NA, not applicable; these variables were used to match cases and controls.

Data are presented as number (%) unless indicated otherwise.

a The onset of bacteraemia for 27 of 235 cases was within 2 days post-hospital admission; for 25 of 27 of these cases there was healthcare contact in the preceding 3 months.

b Lead specialties included 18 different options used for matching, grouped under six categories in this table. A full list of specialties is shown in the Supplementary data.

c Other species were *Enterococcus raffinosus* (1), mixed *E. faecalis* and *E. faecium* (1), mixed *E. faecium* and *E. raffinosus* (1) and one unspeciated. Both *E. raffinosus* isolates were phenotypically VanA.

d Fisher’s exact test.

A univariable analysis was performed to identify risk factors associated with VRE bacteraemia (Table 2 and Table S2). This demonstrated associations with the following: cumulative length of stay on high-risk wards at CUH during the year preceding the current admission; inpatient transfer from another hospital; gastric acid-suppression therapy; presence of a CVC; neutropenia; solid organ tumour; severe renal failure; gastrointestinal disease; hepatobiliary disease; diabetes with end-organ damage; and hypoalbuminaemia.

**Table 2.** Risk factors for VRE bacteraemia

Variable	Cases (*n *=* *235)	Controls (*n *=* *235)	Crude cOR (95% CI)	*P*
Comorbidities				
solid organ tumour	60 (25.5)	42 (17.9)	1.7 (1.0–2.8)	0.03
haematological malignancy	94 (40.0)	86 (36.6)	1.6 (0.8–3.2)	0.17
neutropenia	89 (37.9)	38 (16.2)	6.7 (3.3–13.4)	<0.001
severe renal failure on admission	15 (6.4)	6 (2.6)	3.3 (1.1–10.0)	0.04
liver cirrhosis	29 (12.3)	24 (10.2)	2.0 (0.7–5.9)	0.21
gastrointestinal disease	67/233 (28.8)	25 (10.6)	4.6 (2.5–8.6)	<0.001
hepatobiliary disease	41 (17.5)	22 (9.4)	3.4 (1.5–7.4)	0.003
diabetes (with end-organ damage)	16 (6.8)	5 (2.1)	4.7 (1.3–16.2)	0.02
hypoalbuminaemia	209 (88.9)	150 (63.8)	8.4 (4.0–17.4)	<0.001
Clinical exposures				
gastric-acid suppression	203 (86.4)	183 (77.9)	1.9 (1.1–3.2)	0.01
CVC	197 (83.8)	153 (65.1)	5.4 (2.8–10.6)	<0.001
immunosuppression (other than neutropenia)	146 (62.1)	143 (60.9)	1.2 (0.6–2.1)	0.65
abdominal procedures	85 (36.2)	71 (30.2)	1.6 (1.0–2.8)	0.07
Prior microbiology				
VRE grown from clinical sample within 1 year prior to matching	38 (16.2)	25 (10.6)	1.6 (0.9–2.6)	0.09
Hospital exposure				
cumulative length of stay at CUH within 1 year of current admission (all wards)				
0 days	88 (37.4)	101 (43.0)	1.0	0.16
1–14 days	43 (18.3)	49 (20.9)	1.1 (0.6–1.8)	
>14 days	104 (44.3)	85 (36.2)	1.5 (1.0–2.4)	
cumulative length of stay at CUH within 1 year of current admission (high-risk wards)^a^				
0 days	121 (51.5)	142 (60.4)	1.0	0.05
1–14 days	33 (14.0)	30 (12.8)	1.5 (0.8–2.7)	
>14 days	81 (34.5)	63 (26.8)	1.8 (1.1–3.0)	
ICU stay current admission	87 (37.0)	77 (32.8)	1.6 (0.9–2.9)	0.14
transfer from another hospital	65 (27.7)	44 (18.7)	1.7 (1.1–2.6)	0.02

Data are presented as number (%) of patients.

a High-risk wards included adult and paediatric haemato-oncology, solid organ transplant, nephrology, hepatology and ICU.

The univariable analysis also examined the association between VRE bacteraemia and antibiotic use (Table 3 and Table S2). Both groups had high rates of overall exposure to antibiotics in the preceding 30 days, but cases received antibiotics more often and for longer durations. The commonest antibiotics prescribed in both groups were intravenous vancomycin, meropenem, fluoroquinolones, piperacillin/tazobactam and metronidazole. We found an association with VRE bacteraemia for cumulative antibiotic duration over the prior 30 days and for exposure to intravenous vancomycin, meropenem, cephalosporins, fluoroquinolones, aminoglycosides, penicillins and antifungals. The duration of exposure to intravenous vancomycin, meropenem, fluoroquinolones, cephalosporins and antifungals was also associated with VRE bacteraemia.

**Table 3.** Association between antimicrobial exposure and VRE bacteraemia

	Cases (*n *=* *235)	Controls (*n *=* *235)	Crude cOR (95% CI)	*P*
Antimicrobial				
vancomycin (intravenous)	169/233 (72.5%)	123/234 (52.6%)	3.3 (2.0–5.4)	<0.001
vancomycin (oral)	10/233 (4.3%)	8/234 (3.4%)	1.3 (0.5–3.2)	0.64
cephalosporins	33/233 (14.2%)	18/234 (7.7%)	2.2 (1.1–4.2)	0.02
fluoroquinolones	144/234 (61.5%)	100/234 (42.7%)	2.5 (1.6–3.8)	<0.001
amoxicillin/clavulanic acid	36/233 (15.5%)	31/234 (13.3%)	1.2 (0.7–2.1)	0.49
piperacillin/tazobactam	74/233 (31.8%)	63/234 (26.9%)	1.4 (0.8–2.3)	0.21
meropenem	157/233 (67.4%)	109 (46.4%)	2.8 (1.8–4.3)	<0.001
metronidazole	70/234 (29.9%)	54/234 (23.1%)	1.5 (1.00–2.4)	0.07
aminoglycosides	53/233 (22.8%)	35/234 (15.0%)	2.00 (1.1–3.3)	0.02
penicillins	50/233 (21.5%)	36/234 (15.4%)	1.8 (1.0–3.0)	0.05
macrolides	31/233 (13.3%)	42 (17.9%)	0.7 (0.4–1.2)	0.16
antifungals	167/233 (71.7%)	128 (54.5%)	3.0 (1.8–5.1)	<0.001
Antimicrobial duration				
vancomycin (intravenous)				
none	64/233 (27.5%)	111/234 (47.4%)	1.0	<0.001
1–3 days	28/233 (12.0%)	40/234 (17.1%)	1.5 (0.8–2.9)	
4–7 days	49/233 (21.0%)	37/234 (15.8%)	3.2 (1.6–6.0)	
>7 days	92/233 (39.5%)	46/234 (19.7%)	5.4 (2.9–10.0)	
fluoroquinolones				
none	90/232 (38.8%)	134/233 (57.5%)	1.0	<0.001
1–3 days	32/232 (13.8%)	27/233 (11.6%)	2.0 (1.1–3.7)	
4–7 days	39/232 (16.8%)	25/233 (10.7%)	2.6 (1.4–4.8)	
>7 days	71/232 (30.6%)	47/233 (20.2%)	2.7 (1.6–4.7)	
meropenem				
none	76/233 (32.6%)	126/231 (54.6%)	1.0	<0.001
1–3 days	27/233 (11.6%)	18/231 (7.8%)	2.6 (1.3–5.4)	
4–7 days	39/233 (16.7%)	35/231 (15.2%)	2.1 (1.2–3.8)	
>7 days	91/233 (39.1%)	52/231 (22.5%)	4.3 (2.4–7.7)	

Only risk factors found to be statistically significant on multivariable analysis are shown.

Factors that were significant in the univariable analysis were then used in a multivariable analysis to define independent risk factors for VRE bacteraemia (Table 4). After adjustment for comorbidities, when compared with patients who did not receive any intravenous vancomycin, those exposed for 1–3 days, 4–7 days or more than 7 days had a stepwise increase in the risk of developing VRE bacteraemia [cOR of 1.2 (95% CI 0.4–3.8), 3.8 (95% CI 1.2–11.7) and 6.6 (95% CI 1.9–22.8), respectively]. Similar stepwise increases in cORs were observed for fluoroquinolones and meropenem. Additional risk factors independently associated with an increased risk of VRE bacteraemia were: presence of a CVC [cOR 8.7 (95% CI 2.6–29.5)]; neutropenia [cOR 15.5 (95% CI 4.2–57.0)]; hypoalbuminaemia [cOR 8.5 (95% CI 2.4–29.5)]; solid organ tumour [cOR 4.4 (95% CI 1.6–12.0)]; gastrointestinal disease [cOR 12.4 (95% CI 4.2–36.8)]; and hepatobiliary disease [cOR 7.9 (95% CI 2.1–29.9)].

**Table 4.** Independent risk factors associated with VRE bacteraemia

Variable	Adjusted cOR (95% CI)	*P*
Vancomycin (intravenous) duration (days)		
none	1.0	0.004
1–3	1.2 (0.4–3.8)	
4–7	3.8 (1.2–11.7)	
>7	6.6 (1.9–22.8)	
Fluoroquinolone duration (days)		
none	1.0	<0.001
1–3	1.3 (0.4–3.7)	
4–7	4.5 (1.6–12.9)	
>7	6.9 (2.4–20.0)	
Meropenem duration (days)		
none	1.0	0.03
1–3	1.8 (0.5–6.4)	
4–7	2.3 (0.8–6.3)	
>7	3.5 (1.3–10.0)	
CVC	8.7 (2.6–29.5)	0.001
Neutropenia	15.5 (4.2–57.0)	<0.001
Hypoalbuminaemia	8.5 (2.4–29.5)	0.001
Solid organ tumour	4.4 (1.6–12.0)	0.003
Hepatobiliary disease	7.9 (2.1–29.9)	0.002
Gastrointestinal disease	12.4 (4.2–36.8)	<0.001

## Discussion

In this study, we found that receiving intravenous vancomycin, fluoroquinolones or meropenem was each associated with VRE bacteraemia. We also observed that the risk increased considerably with longer durations of antibiotic exposure (exceeding 3 days and 7 days) for each of these three agents and that the effect was independent of other risk factors. To our knowledge, our study is the largest to investigate risk factors for VRE bacteraemia and the first to have been performed in the UK, in a setting with high levels of VRE endemicity similar to the situation in the USA. These results not only demonstrate an association for these high-risk antibiotics, but also provide a clinically important message, encouraging the discontinuation of these agents within 48–72 h of initiation, when appropriate, to minimize the risk of VRE bacteraemia.

The multivariable model identified previously reported markers of disease severity that predispose to VRE bacteraemia (hypoalbuminaemia, neutropenia and gastrointestinal disease).^16^^,^^18^^,^^23^ It also identified hepatobiliary disease as an independent risk factor, which has not previously been distinguished from gastrointestinal disease. These conditions are likely to predispose a colonized patient to invasive disease through gut or biliary translocation and suggest that patients who develop VRE bacteraemia represent a subgroup of patients with more significant comorbidities than matched controls in the same wards and specialties, irrespective of length of stay or nursing in ICU. The association between a CVC and VRE bacteraemia has been reported previously and could represent a marker of severity of illness or a potential portal for infection.^18^

The role of vancomycin in promoting VRE acquisition is controversial and reported associations, or lack thereof, could be explained by study design. A meta-analysis of early studies investigating the role of vancomycin in hospital-acquired VRE colonization or infection attributed strong associations to confounding by length of stay, control group selection and publication bias.^29^ This goes against human experimental evidence in which administration of glycopeptides orally led to gastrointestinal selection of VRE.^30^ Two studies of VRE bacteraemia using controls without enterococcal bacteraemia have implicated vancomycin exposure as an independent risk factor,^31^^,^^32^ but two further recent studies with adequate sample size failed to demonstrate this effect.^18^^,^^23^ Both of the latter studies were conducted in Australia where vancomycin resistance was predominantly mediated by the *vanB* operon.^6^ This contrasts with the CUH and UK epidemiology where vancomycin resistance in VRE bacteraemia is predominantly mediated by *vanA*.^33^^,^^34^

Carbapenem use has only been implicated as an independent risk factor for VRE compared with VSE bacteraemia in one published study.^14^ However, since VRE was predominantly caused by ampicillin-resistant vancomycin-resistant *E. faecium* and VSE by ampicillin-susceptible vancomycin-susceptible *Enterococcus faecalis*, the effect could have been previously overestimated.^14^ This is particularly the case as imipenem, which was the carbapenem used in this study, has higher efficacy against ampicillin-susceptible enterococci compared with other carbapenems. Our study supports the independent association of carbapenem (meropenem) use with VRE bacteraemia. Carbapenems have anaerobic activity, which could promote VRE colonization.^35^ A number of investigators have reported that antibiotics with anaerobic activity predispose to VRE colonization,^9^ but definitions of this group of antibiotics have not been applied consistently in the literature. Interestingly, piperacillin/tazobactam, an antibiotic with a similar spectrum of activity to meropenem, including anaerobic, was not associated with VRE bacteraemia here. This is consistent with murine experiments in which administration of piperacillin/tazobactam was protective against the establishment of high-level VRE colonization^36^ and with some observational studies in which antimicrobial stewardship interventions involving replacement of cephalosporin use with piperacillin/tazobactam resulted in reduction of VRE colonization.^11^ However, this effect was not noted by other investigators.^37^ It is possible that meropenem was preferentially used in sicker patients in our study or that the lack of observed association with piperacillin/tazobactam was owing to insufficient power. The impact of switching therapy from meropenem to piperacillin/tazobactam on the acquisition of VRE infection merits further investigation.

Fluoroquinolone use has often been identified as a risk factor for VRE bacteraemia on univariable analysis but not following adjustment for other factors.^14^^,^^18^^,^^21^^,^^23^ In a meta-analysis of 10 studies reported by Harbarth *et al.*,^38^ fluoroquinolone use was associated with VRE colonization or infection (pooled OR 2.33, 95% CI 1.5–3.61). In a recent prospective observational study, Sánchez-Díaz *et al.*^39^ showed that long-term prophylaxis with levofloxacin in neutropenic haemato-oncology patients led to intestinal overgrowth of hospital-adapted clones of *E. faecium*.

Placing our findings into the context of the published literature, longer courses of fluoroquinolones and meropenem may promote gut colonization with hospital-adapted strains of *E. faecium* (VSE or VRE depending on local epidemiology). Plausibly, in settings where VRE is endemic, intravenous vancomycin could shift the balance of the gut population and/or invasive isolates from VSE to VRE^40^ thus increasing the risk of VRE bacteraemia in susceptible patients. Gastrointestinal or hepatobiliary insults or presence of a CVC could constitute portals of entry for the infection in heavily colonized patients, particularly those with neutropenia.

This study has a number of limitations. It was conducted in a single centre with high endemicity of VRE and so the findings may not apply to other settings, particularly those that utilize active screening programmes for VRE. However, the infection control practices in our hospital are typical for the UK and the study included patients from all high-risk groups including adult and paediatric populations. Thirty-eight patients with positive blood cultures for VRE, considered a contaminant based on contemporaneous clinical assessment, were excluded. These patients had a comparable 30 day mortality to the control population (8%) and repeat blood cultures performed in 33 patients (87%) were negative in the absence of treatment supporting their exclusion. Cases and controls differed in the duration of prior hospitalizations both at CUH and elsewhere and, despite adjusting for these in the model, there could be residual confounding. We did not adjust for the Charlson comorbidity index as this score is not applicable to children, but analysed its individual components instead. Also, we did not adjust for acute severity of illness using the Pitt bacteraemia or other scores, as we could not ascertain whether the observed score was a cause or an effect of the bacteraemia. We opted against using a case-case-control design which has been advocated for studies of antibiotic resistance to enable distinction between risk factors predisposing to infection by a particular organism as opposed to those specific to its resistance marker.^41^ This decision was made because VRE bacteraemia tends to occur later in the course of hospitalization than VSE bacteraemia,^18^^,^^23^ so accounting for markers of hospital exposure related to length of stay would not have been possible. Consequently, some of the findings should be interpreted as potentially predisposing to both VSE and VRE bacteraemia rather than just VRE as explained above.

In conclusion, this study identified longer duration of exposure to vancomycin, fluoroquinolones or meropenem as independent risk factors for VRE bacteraemia. Antibiotic formulary interventions have not been proven to be effective in reducing VRE bacteraemia but are generally accepted as part of a response to curb resistant pathogens, in addition to infection control interventions such as handwashing and improved cleaning protocols.^42^^,^^43^ This study suggests that targeting the use of a single antibiotic in an endemic setting is unlikely to significantly affect rates of VRE bacteraemia. Instead, a combination of approaches including antimicrobial stewardship focusing on limiting the duration of high-risk antibiotics in addition to infection control interventions would be required to curb the rates of VRE bacteraemia.

## Supplementary Material

Supplementary Data
