# Supplementary material for: Duration of exposure to multiple antibiotics is associated with increased risk of VRE bacteraemia: a nested case-control study
Source: J Antimicrob Chemother. 2018 Mar 13;73(6):1692–9. doi: 10.1093/jac/dky075 (PMC5961253; doi:10.1093/jac/dky075)
Supplement: Supplementary Data [file dky075_supplementary_data.docx]

**Supplementary data**

**Matching**

A full list of specialties used for matching included: colorectal surgery, gastroenterology, general medicine, general surgery, geriatrics, haematology, hepatology, hepatobiliary and pancreatic surgery, infectious diseases, liver transplant, medical oncology, nephrology, neurology, neurosurgery, paediatric haemato-oncology, renal transplant, small bowel transplant, thoracic medicine, upper gastrointestinal surgery, and urology.

**Definitions**

Gastrointestinal disease was defined as one or more of the following: bowel obstruction, pseudo-obstruction or ileus; gastrointestinal graft versus host disease; enteric fistula; gastrointestinal ischemia or necrosis; perforation of oesophagus, stomach or bowel, including anastomotic leak; *Clostridium difficile* associated colitis or toxic megacolon (assessed radiologically or by colonoscopy in addition to positive microbiological diagnosis); inflammatory colitis; neutropenic enterocolitis/typhlitis; other colitis (such as cytomegalovirus). Hepatobiliary disease was defined as one or more of the following: biliary stricture, obstruction, stent or percutaneous transhepatic cholangiogram catheter; bile leak; primary sclerosing cholangitis; cholecystitis or gallbladder empyema; pancreatitis. Severe renal disease was defined as i) pre-existing requirement for renal replacement therapy prior to admission; or ii) persistent serum creatinine >265 μmol/L (3 mg/dL) prior to admission. Abdominal procedures within 30 days prior to matching, included abdominal surgery, percutaneous aspiration or drainage of ascites or abdominal collections, colonoscopy, flexible sigmoidoscopy or gastroscopy. Immunosuppression included receipt of systemic cytotoxic chemotherapy within the last 30 days, systemic steroid use (defined as the equivalent of >5 mg prednisolone orally per day), systemic calcineurin inhibitors or other immunosuppressive agents (including azathioprine, mycophenolic acid, cyclophosphamide and biologic agents). Cephalosporins included ceftriaxone, ceftazidime, and cefalexin. Fluoroquinolones included ciprofloxacin and moxifloxacin. Penicillins included penicillin V or G, flucloxacillin, and amoxicillin. Macrolides included clarithromycin, erythromycin, and azithromycin. Miscellaneous agents included doxycycline, rifampicin, nitrofurantoin, trimethoprim, fusidic acid, tigecycline, and daptomycin.

The Charlson comorbidity index (1) was calculated at the time of admission and the Pitt bacteraemia score (2) was calculated as the highest score within 48 hours prior to matching, but these scores were not included in the multivariable analysis as explained in the discussion.

**References**

1. Charlson ME, Pompei P, Ales KL, MacKenzie CR. A new method of classifying prognostic comorbidity in longitudinal studies: development and validation. *J Chron Dis* 1987; **40**:373-83.

2. Chow JW, Yu VL. Combination antibiotic therapy versus monotherapy for gram-negative bacteraemia: a commentary. *Int J Antimicrob Agents* 1999; **11:**7-12.

**Table S1.** Characteristics of 38 patients excluded due to presumed contamination

| **Characteristic** | **Excluded patients (n=38)** |
| --- | --- |
| Age (years), median (IQR) | 59.5 (52-70 |
| Male | 21 (55.3) |
| Year of admission |  |
| 2005 | 3 (7.9) |
| 2006 | 6 (15.8) |
| 2007 | 5 (13.2) |
| 2008 | 9 (23.7) |
| 2009 | 5 (13.2) |
| 2010 | 3 (7.9) |
| 2011 | 4 (10.5) |
| 2012 | 3 (7.9) |
| Ward at time of positive blood culture |  |
| Adult general | 29 (76.3) |
| Adult ICU | 5 (13.2) |
| Paediatric | 4 (10.5) |
| Time from admission to positive blood culture (days), median (IQR) | 19 (14-29) |
| Lead specialty type at time of positive blood culture |  |
| Adult haematology | 14 (36.8) |
| Adult oncology | 1 (2.6) |
| Adult medicine | 14 (36.8) |
| Adult solid organ transplant | 2 (5.3) |
| Adult surgery | 3 (7.9) |
| Paediatric haemato-oncology | 2 (5.3) |
| Neonatology | 2 (5.3) |
| *Enterococcus faecalis* | 2 (5.3) |
| *Enterococcus faecium* | 32 (84.2) |
| Other enterococcal species^a^ | 4 (10.5) |
| Death within 30 days of positive blood culture | 3 (7.9) |

^a^ *E. raffinosus* (n=2) and unspeciated (n=2).

**Table S2.** Risk factors for vancomycin-resistant enterococcal bacteraemia

| **Variable** |  | **Cases (n=235)** | **Controls (n=235)** | **Crude cOR (95% CI)** | ***P* value** |
| --- | --- | --- | --- | --- | --- |
| Age (years), median (IQR) |  | 56.6 (39.0-66.7) | 57.2 (38.4-68.7) | 1.0 (1.0-1.01) | 0.34 |
| Sex (male) |  | 145 (61.7) | 137 (58.3) | 1.1 (0.8-1.6) | 0.47 |
| **Ward Stay** |  | | | | |
| ICU stay current admission (days), mean (SD) |  | 4.1 (±9.7) | 4.4 (±11.9) | 1.0 (1.0-1.0) | 0.56 |
| **Antibiotic Exposure** |  | | | | |
| Any antibiotic^a^ |  | 231/234 (98.7) | 214 (91.1) | 19 (2.5-141.9) | 0.004 |
| Cumulative antibiotic duration | 0-7 days | 40/232 (17.2) | 76/234 (32.5) | 1.0 | <0.001 |
|  | 8-14 days | 61/232 (26.3) | 62/234 (26.5) | 2.2 (1.2-4.0) |  |
|  | 15-30 days | 131/232 (56.5) | 96/234 (41.0) | 4.4 (2.3-8.5) |  |
| Cumulative antibiotic duration, median (IQR) |  | 16 (11-25) | 11 (5-20) | 1.1 (1.0-1.1) | <0.001 |
| Total number of antibiotics, median (IQR) |  | 4 (3-5) | 3 (2-4) | 1.56 (1.3-1.8) | <0.001 |
| Teicoplanin |  | 9/233 (3.9) | 2/234 (0.9) | 4.5 (1.0-20.8) | 0.05 |
| Linezolid |  | 8/233 (3.4) | 5/234 (2.1) | 1.6 (0.5-4.9) | 0.41 |
| Trimethoprim-sulfamethoxazole |  | 58/233 (24.9) | 56/234 (23.9) | 1.1 (0.7-1.9) | 0.69 |
| Clindamycin |  | 2/233 (0.9) | 2/234 (0.9) | 1.0 (0.1-7.1) | 1.00 |
| Miscellaneous^b^ |  | 21/233 (9.0) | 15/234 (6.4) | 1.5 (0.7-3.0) | 0.29 |
| **Antimicrobial Duration** |  | | | | |
| Co-amoxiclav duration | None | 197/232 (84.9) | 203/234 (86.8) | 1.0 | 0.09 |
|  | 1 to 3 days | 23/232 (9.9) | 17/234 (7.3) | 1.4 (0.7-2.9) |  |
|  | 4 to 7 days | 4/232 (1.7) | 11/234 (4.7) | 0.4 (0.1-1.3) |  |
|  | >7 days | 8/232 (3.5) | 3/234 (1.3) | 2.5 (0.7-9.9) |  |
| Piperacillin-tazobactam duration | None | 160/233 (68.7) | 171/233 (73.4) | 1.0 | 0.13 |
|  | 1 to 3 days | 20/233 (8.6) | 27/233 (11.6) | 0.8 (0.4-1.6) |  |
|  | 4 to 7 days | 23/233 (9.9) | 16/233 (6.9) | 1.7 (0.8-3.6) |  |
|  | >7 days | 30/233 (12.9) | 19/233 (8.2) | 1.8 (0.9-3.5) |  |
| Antifungal duration | None | 66/232 (28.5) | 107/233 (45.9) | 1.0 | <0.001 |
|  | 1 to 3 days | 19/232 (8.2) | 14/233 (6.0) | 2.9 (1.3-6.6) |  |
|  | 4 to 7 days | 25/232 (10.8) | 14/233 (6.0) | 3.7 (1.7-8.0) |  |
|  | >7 days | 122/232 (52.6) | 98/233 (42.1) | 2.9 (1.7-5.1) |  |
| **Co-morbidities** |  | | | | |
| Neutropenia duration (days), mean (SD) |  | 6.2 (±18.2) | 2.2 (±7.5) | 1.1 (1.0-1.1) | 0.001 |
| Pitt bacteraemia score, median (IQR) |  | 2 (1-4) | 0 (0-2) | 1.5 (1.3-1.7) | <0.001 |
| Charlson comorbidity index, median (IQR) |  | 3 (2-4) | 2 (2-3) | 1.3 (1.2-1.4) | <0.001 |
| Haematological malignancy |  | 94 (40.0) | 86 (36.6) | 1.6 (0.8-3.2) | 0.17 |
| No haematological malignancy |  | 141 (60.0) | 149 (63.4) | 1.0 | 0.02 |
| Acute leukaemia |  | 63 (26.8) | 42 (17.9) | 2.1 (1.0-4.5) |  |
| Other haematological malignancy |  | 31 (13.2) | 44 (18.7) | 1.0 (0.5-2.3) |  |
| Solid organ transplant |  | 34 (14.5) | 33 (14.0) | 1.5 (0.3-9.0) | 0.66 |
| Liver |  | 22 (64.7) | 20 (60.6) |  |  |
| Kidney |  | 6 (17.7) | 6 (18.2) |  |  |
| Kidney-pancreas |  | 2 (5.9) | 1 (3.0) |  |  |
| Multivisceral or small bowel |  | 3 (8.9) | 3 (9.1) |  |  |
| Other |  | 1 (2.9) | 3 (9.1) |  |  |
| Hematopoietic stem cell transplant |  | 31 (13.2) | 29 (12.3) | 1.1 (0.6-2.3) | 0.72 |
| Allogeneic |  | 20 (64.5) | 22 (78.9) |  |  |
| Autologous |  | 10 (32.3) | 6 (21.4) |  |  |
| Cord blood |  | 1 (3.2) | 0 (0) |  |  |
| Peptic ulcer disease |  | 20 (8.5) | 6 (2.3) | 3.8 (1.4-10.2) | 0.008 |
| Previous myocardial infarction |  | 11 (4.7) | 15/234 (6.4) | 0.7 (0.3-1.6) | 0.42 |
| Peripheral vascular disease |  | 16 (6.8) | 7 (3.0) | 2.5 (1.0-6.4) | 0.06 |
| Chronic lung disease |  | 41 (17.5) | 34 (14.5) | 1.3 (0.8-2.1) | 0.36 |
| Diabetes (without end-organ damage) |  | 18 (7.7) | 23 (9.8) | 0.7 (0.3-1.5) | 0.34 |

Data are presented as number (%) of patients unless indicated otherwise.

Abbreviations: CI, confidence intervals; cORs, conditional odds ratios; IQR, interquartile range; SD, standard deviation.

^a^ Only includes antibacterial agents.

^b^ Includes doxycycline, rifampicin, nitrofurantoin, trimethoprim, fusidic acid, tigecycline, daptomycin.
